# Penalized canonical correlation analysis reveals a relationship between temperament traits and brain oscillations during mind wandering

**DOI:** 10.1002/brb3.3428

**Published:** 2024-02-15

**Authors:** Erkka Heinilä, Aapo Hyvärinen, Lauri Parkkonen, Tiina Parviainen

**Affiliations:** ^1^ Faculty of Information Technology University of Jyväskylä Jyväskylä Finland; ^2^ Department of Computer Science University of Helsinki Helsinki Finland; ^3^ Université Paris‐Saclay, Inria, CEA Gif‐sur‐Yvette France; ^4^ Department of Neuroscience and Biomedical Engineering Aalto University School of Science Espoo Finland; ^5^ Centre of Interdisciplinary Brain Research, Department of Psychology, Faculty of Education and Psychology University of Jyväskylä Jyväskylä Finland

**Keywords:** anxiety, canonical correlation analysis, magnetoencephalography, mind wandering, mindfulness

## Abstract

**Introduction:**

There has been a growing interest in studying brain activity under naturalistic conditions. However, the relationship between individual differences in ongoing brain activity and psychological characteristics is not well understood. We investigated this connection, focusing on the association between oscillatory activity in the brain and individually characteristic dispositional traits. Given the variability of unconstrained resting states among individuals, we devised a paradigm that could harmonize the state of mind across all participants.

**Methods:**

We constructed task contrasts that included focused attention (FA), self‐centered future planning, and rumination on anxious thoughts triggered by visual imagery. Magnetoencephalography was recorded from 28 participants under these 3 conditions for a duration of 16 min. The oscillatory power in the alpha and beta bands was converted into spatial contrast maps, representing the difference in brain oscillation power between the two conditions. We performed permutation cluster tests on these spatial contrast maps. Additionally, we applied penalized canonical correlation analysis (CCA) to study the relationship between brain oscillation patterns and behavioral traits.

**Results:**

The data revealed that the FA condition, as compared to the other conditions, was associated with higher alpha and beta power in the temporal areas of the left hemisphere and lower alpha and beta power in the parietal areas of the right hemisphere. Interestingly, the penalized CCA indicated that behavioral inhibition was positively correlated, whereas anxiety was negatively correlated, with a pattern of high oscillatory power in the bilateral precuneus and low power in the bilateral temporal regions. This unique association was found in the anxious‐thoughts condition when contrasted with the focused‐attention condition.

**Conclusion:**

Our findings suggest individual temperament traits significantly affect brain engagement in naturalistic conditions. This research underscores the importance of considering individual traits in neuroscience and offers an effective method for analyzing brain activity and psychological differences.

## Graphical abstract

In the article, we present a novel way to jointly analyze brain oscillations and temperament characteristics with penalized canonical correlation analysis in a clinically relevant context of mindful and anxious states of minds. The main results include the specific regions of the brain where the brain oscillations are modulated by the temperament traits in anxious situations, and the specific regions where the brain correlates of focused attention differ from the brain correlates of mind wandering.

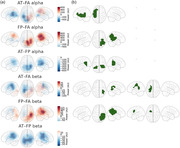

## 1 INTRODUCTION

Studies using functional magnetic resonance imaging (fMRI) and, recently, also magnetoencephalography (MEG) have shown that even with no specific stimulus, the brain exhibits robust patterns of activation that are consistent over individuals (Beckmann et al., 2005; Brookes et al., 2011; Nugent et al., 2015; Ramkumar et al., 2012). Although it is important to study what is common over individuals, for better understanding the relevance of naturalistic brain dynamics in individual experience and behavioral tendencies, we also need to explore how individual brains consistently differ in these conditions. Indeed, with an increase in sample size and the use of more sophisticated data analysis methods, it is becoming feasible to extract features in brain activation patterns that are associated with individual trait differences in cognition or behavior (Mason et al., 2007). For example, the frequency distribution of the spectral content of resting state electrophysiological activity in the brain shows stability over individuals (Näpflin et al., 2007) and reflects the level of cognitive load (Haegens et al., 2014).

There are very few investigations that would have focused on the link between oscillatory dynamics in naturalistic conditions and psychological traits within a typical healthy sample of participants. Interestingly, MEG was recently used to show that increased beta‐band modulation to anxiety‐evoking images differs between participants who profile at the opposite ends in the dimension of tendency to be exploratory versus tendency to be cautious (Yamano et al., 2016). In that study, the differences were shown at the group level by classical hypothesis‐driven *t*‐statistics on activation in response to stimulation. Yet, the results indicate that there are indeed features in the MEG oscillations that may reflect, in a fundamental way, how we approach the environment, especially in emotionally loaded contexts.

When seeking to uncover features in brain activation patterns relevant to individual psychological characteristics, dispositional traits grounded in neurobiology emerge as the primary dimensions for investigation. For instance, the behavioral approach style (behavioral inhibition vs. approach) is deeply anchored in neuroanatomy and has received empirical support from animal studies (Cavigelli et al., 2007). Specifically, the behavioral approach style is linked to regions like the left prefrontal cortex (PFC) and the ventral striatum. In contrast, behavioral inhibition corresponds with the right PFC and the amygdala (Davidson et al., 2000; Harmon‐Jones & Allen, 1997). On a broader scale, affective tendencies, including anxiety and depressive mood, can be associated with various neurobiological mechanisms, such as the dynamics of certain neurotransmitters (Harmer et al., 2009).

Previous studies conducted with fMRI have provided valuable information about the cortical networks related to different cognitive states. The moments of mind wandering have been associated with increased activation in the so‐called default mode network (DMN), primarily consisting of posterior cingulate cortex/precuneus, medial PFC, and bilateral angular gyrus (Fox et al., 2015). In the beginning, this network was mainly considered to be a correlate of not executing any task but has since been associated also with active processing such as self‐referential thought and social cognition (Mars et al., 2012). On the other hand, the frontoparietal control network (FPN), which consists of posterior parietal cortices and dorsolateral PFC, has been associated with focused attention (FA) (Kajimura et al., 2020; Marzetti et al., 2014; Taren et al., 2017). FPN is often yet divided into dorsal and ventral streams, with the dorsal attention network consisting of the intraparietal sulcus and the frontal eye fields, responsible for the top–down intentional tasks, and the ventral attention network consisting of the temporoparietal junction (TPJ) and the inferior‐to‐middle frontal gyrus (IFG, MFG), responsible with directing attention to salient stimuli (Mengotti et al., 2020). Moreover, decreases in the activation of DMN have been witnessed during FA meditation when compared to resting state condition (Garrison et al., 2015).

Electrophysiological investigations, largely conducted using electroencephalography (EEG), have sought to identify the neural correlates underpinning various mental states, including FA meditation and the related mind wandering. These studies have generated diverse results, possibly due to the substantial variation in experimental designs—contrasting different mental states or comparing groups with differing levels of meditation expertise—and the range of meditation styles explored (Deolindo et al., 2020). Many have observed enhanced alpha power in FA meditation, particularly when contrasted with a resting state. However, the results have not always been consistent, with some reporting no effect, or even a decrease in alpha power (DeLosAngeles et al., 2016; Lomas et al., 2015). Some of this discrepancy could potentially be attributed to the lower spatial resolution of EEG, particularly in older studies, and the variability in the resting state condition across individuals and studies. Leveraging the improved spatial accuracy of MEG and the more well‐defined contrasts should facilitate a more robust identification of the differences between FA and mind‐wandering conditions in oscillatory dynamics.

Recent studies utilizing MEG and EEG have clarified the role of different neural oscillations in supporting different cognitive and psychological functions (Cebolla & Cheron, 2019; Lopes da Silva, 2013). Alpha oscillations are generally considered to be involved in the routing of information and in the engagement of task‐relevant neuronal ensembles. Indeed, in the visual domain, the increase in the power of alpha oscillations in one cortical area is thought to indicate active inhibition of that area to allow undisturbed processing in the correct cortical processing pathway (Zumer et al., 2014). The idea has been further established in other sensory domains (Haegens et al., 2011; Weisz et al., 2011) and has been suggested to apply even more broadly, for example, in FPN (Misselhorn et al., 2019). The decrease in the power of alpha oscillations signifies the release of inhibition and can often be thought to be a correlate of cortical activation (Foxe & Snyder, 2011; Klimesch, 2012). Beta oscillations are traditionally associated with sensorimotor processing, where the dynamics often resemble the dynamics of alpha oscillations, that is, they decrease with activation and increase with inhibition. It has been even suggested that alpha and beta might be generated by a common mechanism (Carlqvist et al., 2005). However, recently, modulation of beta band oscillations has been reported in many other areas of the brain, and it has been suggested to have a functional role, for example, in visual perception (Piantoni et al., 2010), language processing (Weiss & Mueller, 2012), and working memory (Siegel et al., 2009). Especially the higher range of beta (20–30 Hz) has been witnessed to increase with mental activation and decrease with inhibition, thus making it possible that beta to certain extent may act independently of alpha (Spitzer & Haegens, 2017).

In this study, we aimed to explore how individual temperament characteristics shape the way the brain behaves in naturalistic conditions. We recorded the brain activity of 28 participants using MEG as they performed 3 distinct tasks. Apart from the FA task, we introduced two conditions that were crafted to simulate aspects of mind wandering, albeit in a structured manner. These conditions required participants to engage in self‐focused, future‐oriented thinking and contemplation on negative, anxiety‐inducing content, respectively. Previous research predominantly identifies mind wandering through probes (e.g., “was your mind wandering just now?”) or self‐detection by the participants (e.g., “if you notice your mind wandering, press a button”) (Weinstein, 2018). However, to maximize the duration of the mind‐wandering data collected and to create clear emotional contrasts for FA—neutral and anxious—we adopted a different approach. It is important to note that the conditions we introduced might be more aptly described as instances of “busy mind,” characterized by engagement with a range of thoughts and feelings, unlike the relatively “empty” state pursued during meditation.

Although these conditions necessitate task‐oriented attention, they are designed to induce a state of controlled or deliberate mind wandering—an effortful, intentional engagement of unguided thoughts (Arabacı & Parris, 2018), akin to the concept of “focused daydreaming” (Dorsch, 2015), where individuals actively direct their thoughts around a particular theme or subject. Although this differs from spontaneous mind wandering, which is typically characterized by unintentional drifts of thought, it still embodies central features commonly associated with mind wandering, such as autobiographical thinking and rumination.

Traditionally, the assessment of interindividual differences in naturalistic conditions has primarily relied on unstructured resting state data, a task that has proven to be notably challenging (see, e.g., Dubois et al., 2018). In our study, we induce mental states that may align with specific psychological traits. For instance, our anxious mind state condition could potentially amplify trait anxiety, thereby rendering it more discernible in the brain activation patterns. We propose that this method may enhance the sensitivity of our analysis in detecting individual differences. By manipulating states, we can underscore trait‐specific characteristics in the brain, thereby offering a more dynamic and context‐dependent perspective on individual differences.

To study the three naturalistic psychologically relevant conditions and their relationship to the dispositional traits, we investigated the oscillatory patterns in the alpha (∼10 Hz) and the beta (∼20 Hz) bands. These bands were chosen as they more often show robust peaks in the spectral density and have previously been shown to be modulated by trait characteristics. For example, Yamano et al. (2016) have shown that beta power response depends on exploratory tendencies of individuals, and Zeev‐Wolf et al. (2018) have shown that the powers of alpha and beta oscillations differ between neurotypical individuals and individuals with schizophrenic symptoms. The analysis was based on spatial contrast maps, which are band‐ and participant‐specific source space representations of the difference between two conditions. When using contrast maps (instead of investigating the activation of one condition at a time), the other condition works as a baseline, and thus noise is removed, and participants are more comparable to each other. We used permutation cluster tests to investigate if the contrast maps had any consistent, significant patterns over participants.

We were specifically interested in exploring the variability in the oscillatory engagement across different mental conditions in the between‐participant domain and its relation to the behavioral trait characteristics of the participants. To accomplish this, we utilized a data‐driven approach using canonical correlation analysis (CCA). This method, especially when penalized with both L1‐ and L2‐norms, provides a robust framework for investigating the relationship between different mental state pairs and behavioral traits and remains effective even with a relatively small sample size.

## 2 MATERIALS AND METHODS

### 2.1 Participants and data acquisition

#### 2.1.1 Imagery stimuli

The imagery used in the conditions came from the International Affective Picture System (IAPS).

We picked 32 anxiety‐inducing negative images (such as images of spiders, accidents, or threatening situations) to be used in the instructions of the anxious thought (AT) condition and 32 neutral images (such as images of hot air balloons, dinner tables, or Christmas trees) to be used in the instructions of the future planning (FP) condition. However, to maximize the relatability and the affective experience, the participants were asked to select 16 out of 32 images that they found they could plan on or that they felt they would feel anxious about for both conditions. The selection was done using a computer before the actual recording sessions started. Thus, for each of the 16 miniblocks of both the AT and the FP conditions, there was a unique instruction image, both to help the participants have relatable and affective experiences and to not run out of ideas. The same picture of clouds in front of a blue sky was always shown for the FA condition.

#### 2.1.2 Participant recruitment and MEG recording

A total of 29 people (aged 21–48 years) took part in the initial data‐gathering phase. Participants had no history of neurological disorders, head trauma, or substance abuse and had normal or corrected‐to‐normal vision. None of the participants were under medication affecting the central nervous system or had psychiatric disorders. A few participants reported that they had experienced depression at some point in life, but no clinical depression was reported at the time of the study. Thirteen participants had no previous meditation experience, whereas other participants had meditation experience ranging from 0.5 to 10 years (for those who had, the mean was 3.4 years, and the standard deviation was 2.4 years). The participants gave written informed consent before any experimental session. The research was conducted according to the ethical principles stated in the Declaration of Helsinki, and the study was accepted by the Ethics Committee of the University of Jyväskylä.

For each of the participants, we collected two 30‐min recordings of MEG with the Elekta Neuromag TRIUX system (MEGIN Oy). Each recording session included two 2‐min resting‐state blocks at the beginning and at the end of the sessions. In between, there were three different 8‐min conditions organized in 2‐min blocks. Each 2‐min block contained two 1‐min miniblocks of the same condition. The 2‐min blocks were organized in a counterbalanced order so that no 2‐min blocks of the same condition were next to each other, and that each condition was not followed by the same condition twice in a row.

**FIGURE 1:**
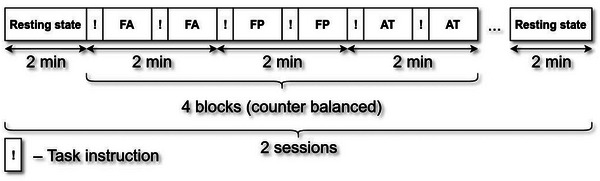
Experimental design. Conditions of focused attention (FA), future planning (FP), and anxious thoughts (AT) are recorded in 2‐min blocks in counterbalanced order. Each 1‐min miniblock contains a brief instruction in the beginning (!). In total, each condition is recorded for 2 sessions × 4 blocks × 2 miniblocks × 1 min/miniblock = 16 min.

The three conditions included FA on breathing, FP, and AT induced by reflection on emotional pictures (AT). In all conditions, participants were instructed to sit still and fix their gaze on a crosshair displayed on the screen, ensuring a consistent visual focal point across all tasks. Prior to each 1‐min miniblock, an instruction image was briefly displayed for 7 s, dictating the respective task: “focus on your breathing” for the FA condition, “make plans related to the picture” for the FP condition, and “place yourself or someone close to you in this situation” for the AT condition. Once the brief instruction period concluded, the image was replaced by the crosshair for the remaining 53 s. After each 2‐min block, participants were queried about their focus and emotional state during the task, responding on scales labeled “Not focused/Focused” and “Unpleasant/Pleasant,” respectively. These responses are detailed in the Supporting Information section (Figures A.9 and A.10) but were not incorporated in the main analysis. Participants were briefed on the session's structure beforehand. Refer to Figure 1 for a visual representation of the experimental design.

### 2.2 Trait questionnaires

Psychological and behavioral traits were characterized using standardized questionnaires. Based on earlier studies (Lyyra & Parviainen, 2018; Schneider et al., 2018; Yamano et al., 2016), we hypothesized that traits along the dimensions of behavioral inhibition and behavioral approach as well as anxiety would most likely be captured in the brain dynamics and, therefore, focused on questionnaires in this domain. These included questionnaires related to anxiety, depression, and behavioral inhibition or activation.

BIS/BAS is a self‐report questionnaire designed to measure two motivational systems: behavioral inhibition system (BIS) and the behavioral activation system (BAS) (Carver & White, 1994). BIS and BAS are proposed as fundamental neurobiologically based motivational systems that regulate approach or withdrawal behaviors (Gray, 1970) and contribute to behavioral choices, affective states, personality, and even psychopathology (Li et al., 2015). BIS score can take a value between 7 and 35, with high values indicating high behavioral inhibition, that is, tendency to withdraw from unfamiliar situations and avoid aversive outcomes, and BAS score can take a value between 13 and 65 with high values indicating high behavioral approach, that is, tendency for novelty seeking and approaching rewarding outcomes. The Beck Depression Inventory (BDI) measures characteristic attitudes and symptoms of depression (Beck et al., 1961), and the score may take a value between 0 and 63, with high values indicating high depression levels. The Beck Anxiety Inventory (BAI) is used for measuring the severity of anxiety in children and adults (Beck et al., 1988), and the score ranges between 0 and 63, with high values indicating high anxiety levels.

### 2.3 Preprocessing of MEG data

MEG was used to record magnetic fields from 306 channels arranged in a helmet around the head of a participant. Recording was done with 1000 Hz sampling frequency, yielding 306 time series, 1 for each channel. Before the recording, the shape of the participant's scalp was digitized, and the five coils attached to the head were localized with respect to anatomical landmarks (nasion, preauricular points). During the MEG measurement, weak current with specified frequency was applied to the coils so that the location of sensors with respect to the helmet could be determined.

The recordings were preprocessed with the signal‐space separation method (MaxFilter 3.0; MEGIN Oy) to suppress magnetic interference originating from outside the helmet. As the head location in the helmet can change during the recording, MaxFilter's utility for movement compensation was used. Common artifacts such as eye blinks and heartbeats were extracted and removed using ICA implemented in MNE‐Python (Gramfort et al., 2014). ICA was run separately for each participant and session (leaving around 30 min of 306‐channel data for a single ICA) and was set up to retain 95% of the original variance. For each ICA, we analyzed the resulting components and selected for removal those that clearly had a topography, spectral signature, and time course of a stereotypic blink or heartbeat component. One participant had to be completely dropped from further analysis because of visibly large noise levels both in time series and spectral densities (possibly due to interference of metal), which we were not able to clean up afterward, leaving 28 participants for the final analysis.

To enhance interpretability, MEG data was transformed to source space using the noise normalized minimum norm estimate method and dynamical statistical parametric mapping (Dale et al., 2000). The noise covariance matrix, used in the procedure, was based on empty room recordings. The source space was a volumetric source dipole arrangement consisting of an equidistant grid of 5218 voxels with 7 mm spacing, and it was based on the average head model from the freesurfer software package. The source space was uniformly scaled (scaling factors varied between 0.85 and 1.0) for each participant separately so that the fit between the digitized head points and the average head would be as good as possible. Both the scaling and fitting were done using MNE‐Python's coregistration utility.

### 2.4 Data analysis

#### 2.4.1 Overview

The overall MEG dataset consisted of 2 independent 8‐min recordings for each of the conditions (FA, FP, and AT) from 28 participants. In the analysis of the data, we aimed to extract two kinds of information: the average brain activation patterns during each condition, and the individual differences among the participants. To achieve these goals, we first transformed the participant‐specific time series data into more manageable spatial contrast maps. The transformation of the data to spatial contrast maps was done separately for both alpha and beta band data and all three different condition pairs: FP–FA, AT–FA, and AT–FP. To analyze the average patterns, we used permutation cluster tests on the spatial contrast maps with participants as samples. To analyze the individual differences, we used CCA for the spatial contrast maps and the psychological trait data. Given the exploratory nature of our research, we have not adjusted for multiple comparisons between different contrasts. For each contrast, however, we employed statistical methods that account for the multiple comparison problem within that contrast, aiming to ensure the robustness and generalizability of our findings.

#### 2.4.2 Spatial contrast maps

To transform time series into spatial contrast maps, we executed the following steps. We computed power spectrums in source space with a 4‐s Hanning window and an overlap of 2 s. The spectrums for each condition were computed for all available time points and were thus based on 16 min of MEG data. The power data under frequency bins specific to the band of interest (for alpha 7–14 Hz; for (high) beta 20–30 Hz) were averaged over frequencies, which resulted in a power map (one power value for each voxel) for each participant, condition, and frequency band. For each participant, the spatial contrast maps for all three condition pairs were computed as a simple subtraction of the conditions of interest, for example, AT–FA contrast was computed as “AT minus FA.”

#### 2.4.3 Average patterns over participants

Given the participant‐specific spatial contrast maps, we used permutation cluster tests to test if the contrast maps had clusters of vertices that were significantly distinct from zero when participants were considered samples. We used the permutation_cluster_test() function from MNE‐python, which is based on the method described by Maris and Oostenveld (2007). This method allows computing a large number of (in our case, spatially) correlated univariate statistical tests and then correcting for the multiple comparisons at a (spatially constrained) cluster‐level. The correlation matrix (with dimensions 5218 × 5218) was used as the metric for adjacency, and the *t*‐test was the statistical test that was computed at every voxel. The 95th percentile of the absolute values of the *t*‐values was used as a threshold for a voxel to be included in a cluster. The final result was based on 1024 permutations.

#### 2.4.4 Canonical correlation analysis

To see if the variation in participant‐specific spatial contrast maps could be explained by psychological traits, we used CCA with the spatial contrast maps as one set of variables and the data from trait questionnaires as the other set of variables. CCA seeks to find such linear combinations of the two sets of variables that the correlation between them would be as high as possible. In our case, finding such statistically significant linear combinations would allow us to interpret that there are areas in the brain where the activation depends on some combination of trait characteristics. As the sample is relatively small, we focus only on the first canonical correlation. Moreover, instead of the most classic formulation of CCA (Hotelling, 1936), a more recent least squares formulation is used (Mai & Zhang, 2019). This makes it possible to add convex penalties for the canonical weights, making the results both more generalizable and more interpretable.

For each of the task contrasts (e.g., AT–FA), CCA was run with two sets of variables. The first set consisted of the task contrast data, that is, spatial contrast maps for both frequency bands and for each participant. The spatial contrast maps of both alpha and beta band data were used in one model to get more explanatory power. The data was preprocessed beforehand with the following steps. First, a rank transformation was used for each variable (2 bands × 5218 voxels) with participants as samples, to make the data more robust against non‐normality and outliers. A rank transformation replaces the values of a variable by their rank in the between‐participants domain. Second, in each band separately, principal component analysis was used to reduce the dimension of the data from 5218 voxels to 4 principal components. The number of dimensions was chosen to be 4 as an empirical compromise of containing enough variance while keeping the number of variables low (see Figure A.11 for a scree plot). Increasing the dimensionality to 5 had a negligible effect on the results. Finally, the band data were combined, and a set of 8 variables for 28 participants resulted. The second set of variables consisted of the four behavioral variables: BDI, BAI, BIS, and BAS. Again, we used rank transformation for each variable, where values were replaced by their rank in the between‐participants domain. This set of variables stayed the same for all three task contrasts.

Given the two sets of variables, CCA essentially constructs, based on the original variables (eight variables for contrast data, and four variables for behavioral data), two new variables, one corresponding to brain variables and the other corresponding to behavioral variables, which have the property of producing the highest correlation coefficient that is possible. It is worth noting that this is different from, for example, finding the highest variance factor for both sets of variables with factor analysis and then computing the correlation coefficient. The factor analysis approach identifies transformations that may or may not be relevant for the association between two sets of variables.

The optimization procedure in CCA is almost guaranteed to produce very high correlation coefficients. Thus, effective precautions need to be taken to avoid false positives in the results. One of the precautions is the dimensionality reduction used for contrast data, as it reduces the amount of combinations that the CCA algorithm has available for use. In fact, the standard CCA does not even work if the amount of variables in either set is higher than the amount of samples. It is good to note, however, that except for the first few highest variance principal components, the variance explained by the components diminishes very quickly. Thus, even though we are losing some data with the dimensionality reduction, the lost data should be of much less importance.

As there are eight brain‐signal variables included into CCA, there will also be eight canonical weights corresponding to the brain variables. The mixing matrix computed in the principal component analysis is our link between the latent space, where the CCA is computed, and the space where the original source space signals are. Thus, the weight maps (in Figures 3 and 4) in Section 3 are reconstructed from these eight canonical weights simply by multiplying the weights with the mixing matrix, which results in weights in the source space. The maps should be interpreted as they seem: they tell us which voxels contribute to the association found with CCA.

#### 2.4.5 Ensuring generalizability

In addition to dimensionality reduction, some other precautions were taken. Instead of using the standard CCA formulation, we used a new formulation that allows adding convex penalties for the weights (Mai & Zhang, 2019). The nature of CCA is such that without penalization, a large amount of variables combined with the noise unavoidably available in the data will almost surely result in very high correlation coefficients, even without any real correlation of interest. Thus, we should either reduce the amount of variables or reduce the size of the solution space.

The brain data was whitened as a preprocessing step, and this reduced the number of variables to just eight, which is small enough given the sample size of 28, and high enough to keep almost all of the variation in the data. However, we can still reduce the solution space using penalization methods. There are two main forms of penalization: penalization that seeks sparse or extreme solutions, and penalization that seeks soft or non‐extreme solutions. The L1‐norm, based on absolute value, leads to a sparse solution that has a lot of zero coefficients with just a few large non‐zero coefficients. The L2‐norm, based on square roots, leads to an opposite kind of non‐extreme solution with most of the coefficients being somewhere in between.

One of the novelties of our study is that we can use different kinds of penalization to different kinds of variable sets. For the trait variables, an L1‐penalty makes sense, as we want the algorithm to prefer sparse solutions. It is easier to interpret the results if we get only one or two non‐zero weights instead of all being non‐zero.

There are some arguments for using the L1‐penalty also for brain signals, as often the brain function localizes spatially to only a small subset of available brain regions. However, as a preprocessing step for CCA, we transformed the source space signals to a latent principal component space, where sparsity does not have the same meaning, and in this space, favoring sparse solutions does not make sense. Yet, favoring non‐extreme solutions in this space might reduce overfitting and allow more generalizable solutions. The utility of the L2 penalization can be tested with cross validation.

We selected the L1‐penalty parameter by hand to a value 0.25, which empirically corresponded with the intuitive goal of leaving only one or two non‐zero variables. Using higher penalty parameter values forces all but one variable to be non‐zero, which is too demanding. Using smaller penalty parameter values does not gain almost any more explanatory power but makes it harder to interpret the model. For the selection of the L2‐penalty parameter, we used stochastic cross validation. We had an equidistant grid of 300 sensible parameter values, and for each of them, we randomly split the data into two 14‐participant samples for 2000 times. In each case, we used the first sample (the training set) to fit the CCA model, and then, using the canonical weights of the first canonical correlation, computed correlation in the other sample (the test set). In the end, the correlation coefficients over the 2000 splits were averaged. This procedure results in a score (the average correlation in the test set) for each L2‐penalty. We then fitted the CCA to the whole sample of 28 participants using the preselected L1‐penalty and the best performing L2‐penalty.

To further assess how robust the results were, we used permutation tests. CCA without regularization was run 2000 times while permuting the participant labels for the brain variables and keeping the labels of the behavioral variables intact. *P*‐value was computed as the number of times the first canonical correlation of a permuted sample was higher than the first canonical correlation from the non‐permuted regularized sample divided by the number of all runs. Note that using the unregularized CCA for the permuted samples ensures (because unregularized version will always result in at least as high correlation coefficient as the regularized one) that the lack of parameter search in the permuted cases does not bias the *p*‐value to be erroneously low. After running the main CCA with eight brain variables, to further elucidate the results from the CCA analysis, we post hoc ran the analysis again but selectively removed sources of variation, for example, we used only data from one frequency band.

## 3 RESULTS

### 3.1 Trait questionnaires

Means, standard deviations, and Spearman correlations of behavioral trait results are shown in Table 1. We used Spearman correlations for robustness, as, for example, BAI was quite skewed due to many low values.

**TABLE 1:** Summary of behavioral trait scores for the 28 participants.

	Mean	Std		BAI	BDI	BIS	BAS
BAI	8.5	6.0	BAI	1.0	0.59***	0.38*	0.02
BDI	6.25	6.3	BDI		1.0	0.34	−0.06
BIS	21.8	5.7	BIS			1.0	−0.25
BAS	49.5	6.4	BAS				1.0

*Note*: In the left panel are the means and the standard deviations for BAI, BDI, BIS and BAS. In the right panel is their Spearman correlation matrix.

Abbreviations: BAI, Beck Anxiety Inventory; BAS, behavioral activation system; BDI, Beck Depression Inventory; BIS, behavioral inhibition system.

*p‐Value for the Spearman correlation coefficient was .044.

***p‐Value for the Spearman correlation coefficient was .0009.

### 3.2 Spatial contrast maps

We computed spatial contrast maps for each participant, for each band of frequencies, and for each pair of conditions. The appendix (Figures A.1–A.6) contains the spatial contrast maps for all participants and for all contrast pairs AT–FA, FP–FA, and AT–FP in the case of alpha power. In all the figures of spatial contrast maps, the red means that the average power of oscillations during the first condition (FP in FP–FA pair) is higher than the average power of oscillations during the second condition (FA in FP–FA pair); the blue means the opposite. Thus, if two areas are under the same color, they are both increasing/decreasing in the first condition relative to the second condition, and if two areas are under different colors, the other one is increasing and the other one is decreasing in the first condition relative to the second condition.

### 3.3 Average patterns over participants

**FIGURE 2:**
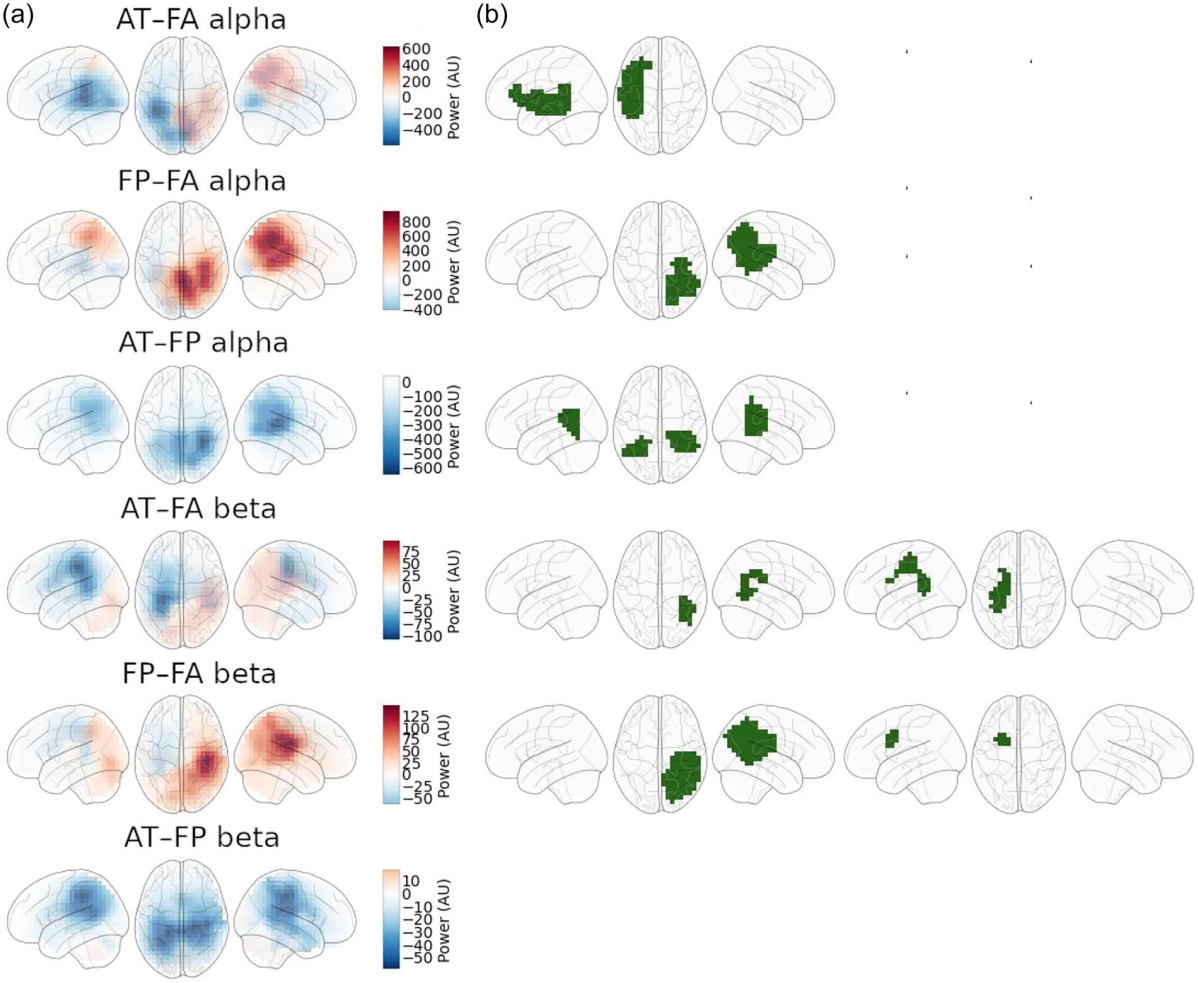
Spatial contrast maps. Maps averaged over participants are shown in panel A, and cluster maps from permutation tests are shown in panel B. Each row corresponds to a specific condition pair in a specific frequency band. Coloring in the left panel corresponds to increase (red) or decrease (blue) of power in the first condition compared to the second condition. In the cluster maps, the green denotes that the area under it belongs to the cluster. Only clusters with *p* < .05 are shown. There can be a cluster for negative or positive values independently; thus, each row can have zero, one, or two significant clusters. Note that the clusters are based on *t*‐values (see Figure A.7) and, for that reason, are not expected to completely align with the mean blobs.

Results at the group level are depicted in Figure 2. Means over participants are shown in panel A, and significant clusters (*p* < .05, not corrected for multiple comparisons) resulting from permutation cluster tests are shown in panel B. The *t*‐values that the cluster permutation tests were based on are shown in Figure A.7. The values in the mean plots get normalized during the inverse transform but are comparable to each other within frequency bands. As the threshold for including voxels in a cluster is somewhat arbitrary, the exact composition and extent of the clusters should be interpreted with caution. Nonetheless, the clusters provide approximate indications of consistent differences and their significance.

Overall, the results for alpha (7–14 Hz) and beta (20–30 Hz) bands are fairly similar to each other in all three condition pairs. Moreover, on average, the difference between the FA and the AT conditions does not notably differ from the difference between the FA and the FP conditions (i.e., AT–FA vs. FP–FA). Roughly speaking, in the right parietal areas, the FP and the AT conditions induced higher alpha and beta activities compared to the FA condition. In contrast, in the left temporal areas, the FP and the AT conditions induced lower alpha and beta activities compared to the FA condition.

A closer examination of the clusters derived from the permutation test shows that for the AT–FA alpha contrast, the difference was localized mainly in the left temporal areas (*p* = .018). For the FP–FA contrast, the parietal areas in the right hemisphere (*p* = .018) were significant. This slight difference between the FP–FA and AT–FA contrasts is seen in the direct comparison of FP and AT, with FP inducing higher alpha power in temporal areas in both hemispheres compared to the AT condition (*p* = .036). In the beta band, a similar right parietal–left temporal pattern emerges with both clusters significant for AT–FA (*p* = .039 for right parietal area; *p* = .033 for left temporal area) and FP–FA (*p* = .028 for right parietal area and *p* = .037 for left temporal area) contrasts. The difference for AT–FP in the beta band was not significant.

### 3.4 Canonical correlation analysis

**FIGURE 3:**
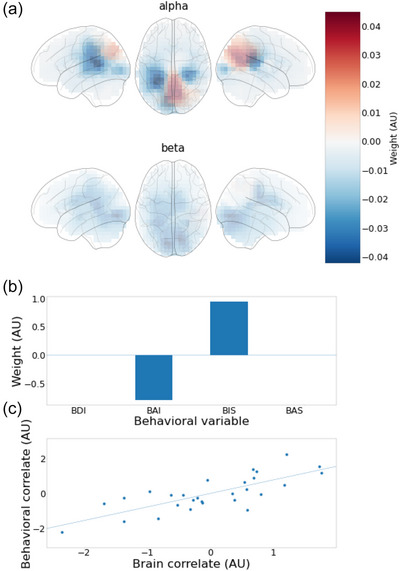
Canonical correlation analysis. The weights and a scatter plot of the only significant canonical correlation (for the condition pair anxious thought–focused attention [AT–FA]) are shown. Panel A shows the weights of brain variables, drawn separately for alpha and beta variables. Panel B shows the weights of behavioral variables. Panel C shows the scatter plot of the two variables constructed from the original variables using the weights in panels A and B. For example, the behavioral correlate was constructed as the linear combination of the scaled behavioral inhibition system (BIS) and Beck Anxiety Inventory (BAI) scores: BIS_weight × (BIS − BIS_mean)/BIS_std + BAI_weight × (BAI − BAI_mean)/BAI_std.

Using the canonical correlation procedure described in Section 2, we investigated if the between‐participants variability in the spatial contrast maps could be correlated with behavioral traits measured with four different questionnaires. With alpha and beta contrast data combined into a single dataset, we ran the analysis for all three different condition pairs and found no significant associations for the FP–FA (*p* > .7) and AT–FP (*p* > .1) contrasts. However, results for the AT–FA contrast were significant (*p* = .015) and are shown in Figure 3.

A specific spatial pattern in the AT–FA contrast map (Figure 3a) showed high correlation (*r* = .75) with a specific combination of trait variables (Figure 3b). In more detail, a pattern of lower alpha power in the temporal areas, especially in the left hemisphere, and higher alpha power in the bilateral precuneus during AT (compared to FA) was correlated (Figure 3c) with a combination of low trait anxiety (BAI) and high behavioral inhibition (BIS).

As the canonical weights were visibly stronger in the alpha than in the beta band, we investigated post hoc whether beta band was necessary for the result by running the analysis again with data only from the alpha band. The results confirm that the alpha band data alone was sufficient for a significant correlation (*p* = .04) with the questionnaire data. When fitting only beta, however, the association diminished and was clearly nonsignificant (*p* > .5), indicating that the connection between the trait variables and brain activity levels was mainly based in the alpha band. Hereafter, we focus only on the alpha band.

For the alpha‐only analysis, the total explained variance of the four variables was 0.85 of the original variance of the 5218 variables. Selection of the L2‐penalty is visually depicted in Figure A.8 and resulted in a non‐zero value, indicating that regularization benefits the analysis.

**FIGURE 4:**
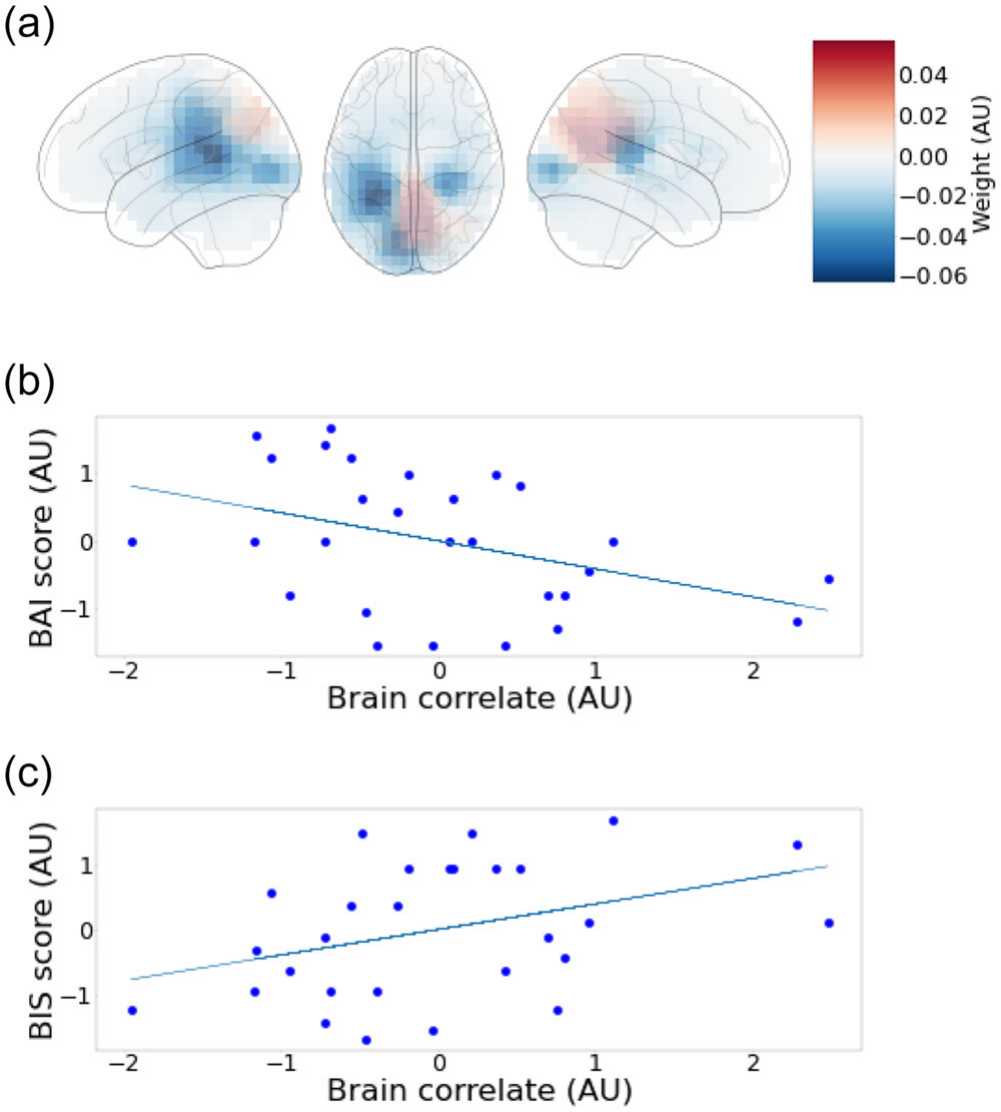
Post hoc analysis. Panel A shows the canonical weights only using alpha‐band data. Panel B shows a scatter plot between Beck Anxiety Inventory (BAI) scores and the brain variable computed with the weights in panel A, whereas panel C shows a scatter plot between behavioral inhibition system (BIS) scores and the same brain variable.

As the anticorrelated pattern of BIS and BAI is somewhat surprising, we further investigated the model parameters. In Figure 4, we show the correlation of the brain variable from the last model (fitted with all behavioral variables, but without beta band) with BAI (Figure 4b) and BIS (Figure 4c) separately. The results show that both of the variables seem to be independently, albeit weakly, associated with the above‐described spatial activation pattern (*r* = −.42 for BAI, *r* = .37 for BIS), but in opposite directions as expected based on the original result. Although the meditation experience (in years) was not incorporated as a covariate in the primary model, we conducted supplementary analyses to investigate its potential influence on the results. Preliminary analyses using a regression approach to control for meditation experience indicated no significant impact on the primary findings (see Figure A.12 for details).

As the AT–FA condition pair is part of both the average pattern results and the CCA results, its main results in the alpha band are summarized in Table 2.

**TABLE 2:** Summary of results of anxious thought‐focused attention (AT–FA) condition pair for both the average patterns and the canonical correlation analysis (CCA).

	Left‐temporal	Precuneus	Right‐parietal	Right‐temporal
Average of AT w.r.t FA	Low	–	–	–
BAI and AT (w.r.t FA)	Correlated	Anticorrelated	–	Correlated
BIS and AT (w.r.t FA)	Anticorrelated	Correlated	–	Anticorrelated

*Note*: “Low” or “high” means that in that area of the brain, alpha power in the AT condition was significantly lower or higher, respectively, than in the FA condition. “Anticorrelated” and “correlated” mean that in that area and against that variable (BAI or BIS), the alpha power in the AT condition with respect to the FA condition is anticorrelated or correlated, respectively, with the variable. For example, in the left‐temporal areas, the higher the BAI score, the higher the (signed) difference between the alpha power of AT and FA conditions.

Abbreviations: BAI, Beck Anxiety Inventory; BIS, behavioral inhibition system.

## 4 DISCUSSION

In this study, we aimed to clarify the oscillatory patterns in the brain during naturalistic conditions and their association with behavioral traits. We analyzed data collected from conditions of FA, self‐centered FP, and AT evoked by visual imagery. Using permutation tests on contrast maps, we found that even with the notable variation across participants, some general patterns emerged. The conditions that simulated “mind wandering,” that is, the FP and the AT conditions, corresponded to higher suppression (higher alpha) of the parietal areas of the right hemisphere compared to the FA condition. Similarly, the FP and the AT conditions corresponded to lower suppression (lower alpha) of the temporal areas of the left hemisphere compared to the FA condition. Although the two “mind wandering” conditions more often evidenced similar patterns relative to the FA condition, stronger suppression of temporal areas in both hemispheres emerged for the contrast between the FP and the AT conditions. To investigate whether the interindividual variability of brain activations across conditions explained individual differences in dispositional characteristics, CCA was used. The only significant association was noted for the alpha power during AT condition relative to FA condition. A pattern of high alpha power in bilateral temporal areas and low alpha power in bilateral precuneus was correlated with BAI scores, representing high tendency to somatic anxiety symptoms, and anticorrelated with BIS scores, mainly representing worry and avoidance of unpleasant situations.

### 4.1 Average patterns over participants

The most apparent difference between the conditions was between the FA condition and the other two, mind‐wandering‐like, conditions. In the mind‐wandering conditions, higher alpha power was evident in the right parietal areas, and lower alpha power was evident in the left temporal areas, when compared to the FA condition. It is not surprising that parietal and temporal areas are modulated with these task demands, as they are considered core areas in the FPN, which is generally associated with control of attention. Interestingly, the patterns resulting in the contrast analysis reflect strong lateralization. However, especially in the ventral stream of FPN, namely, the TPJ and IFG, MFG, right‐dominance has been well established also in earlier studies (Benedek et al., 2014; Kucyi et al., 2012; Mengotti et al., 2020). Furthermore, visuospatial attention has been associated more with the right hemisphere, and for language processing and motor attention, some left‐dominance has been suggested (Caeyenberghs & Leemans, 2014; Mengotti et al., 2020; Ocklenburg & Gunturkun, 2012). It is thus plausible that the oscillatory modulations are not laterally symmetrical, and our results may thus reflect differential balance in the engagement of these functions by the task requirements.

In agreement with our findings, the increased alpha activity in the right hemisphere has been previously associated with creative thinking (Fink & Benedek, 2014; Luft et al., 2018; Mihov et al., 2010), where increased alpha oscillations were thought to be related to inhibition of obvious associations. Indeed, creative thinking has been strongly associated with mind wandering (Fox & Beaty, 2019). Furthermore, parietal regions have been recently linked with prioritizing the focus of attention and cognitive control (Bisley & Goldberg, 2010; Sapountzis et al., 2018). As our conditions required controlling attention, the activation of parietal areas could be interpreted from the perspective of activation of the FPN, which has nodes located in the bilateral parietal areas (Cole et al., 2014). FPN has been suggested to support flexible switching between default and the attentional network, and consequently the focusing of attention to internal, autobiographical information versus external cognition (Smallwood et al., 2012; Spreng et al., 2010).

The lower alpha in the left hemisphere, on the other hand, has not been obviously associated with mind wandering. Rather, the results may reflect increased alpha in these regions during FA. An earlier study, where open monitoring meditation was compared with FA meditation with MEG, reported higher coupling of DMN and parts of FPN in the left hemisphere in the alpha band during FA (Marzetti et al., 2014). Alternatively, but speculatively, it is also possible that the decreased alpha during mind‐wandering conditions reflects language‐related processing. The mind‐wandering conditions, at least when compared to the FA condition, are likely to more strongly engage linguistic associations and verbal content (Bastian et al., 2017). Therefore, the language areas in the left temporal cortex are expected to be less inhibited, as indicated by lower alpha oscillations, when contrasted with FA.

Interestingly, our results also show a bilateral difference in the temporal areas between the AT condition and the FP condition, with AT associated with lower alpha, and presumably less suppression of cortical engagement, in both the left and right hemispheres. The most obvious task‐related difference between these conditions relates to emotional involvement. Although temporal areas have not been directly associated with emotional information processing as such, one may speculate that this could reflect the suggested functional coupling between amygdala and temporal areas (Silverstein & Ingvar, 2015). Indeed, the white matter connectivity via uncinate fasciculus between orbitofrontal and middle temporal areas, including amygdala interactions, has been associated especially with emotional memory (Granger et al., 2021) and anxiety (Lee & Lee, 2020). The increased engagement of these areas during anxious evoking compared to neutral “rumination” could thus, theoretically, relate to a stronger emphasis of emotional memory. An alternative interpretation for the increased activity (decreased alpha power) observed in the parietal regions during the AT condition could relate to the strongly visual essence of the task. Although all the conditions commenced with an instructive image supplemented by text, the AT condition may have inherently necessitated more intense visual processing (Vuilleumier, 2005). The parietal cortices are known, among other functions, for their involvement in higher order visual processing (Konen & Kastner, 2008).

We did not find regions commonly included in the DMN to be more activated in the mind‐wandering states than in the FA, as might be expected based on some earlier findings (Fox et al., 2015; Groot et al., 2021) and based on the general idea of the role of DMN in “task‐free” situations. A potential explanation for this discrepancy could be that our induced mind‐wandering states were not entirely “task‐free,” despite their similarity to natural, unguided thought. Clearly, more research is warranted to achieve better understanding of the specific role of DMN and the way “ongoing free thinking mode” can be defined, both from the brain computational perspective and from the cognitive–psychological perspective. One way forward is to move from “group‐level” interpretations toward individual differences. Indeed, the value of individual differences in better understanding brain–behavior and brain–mind coupling has been recently advocated (Becht et al., 2020; Haegens et al., 2014; Leppäaho et al., 2019).

### 4.2 Canonical correlation analysis

In the present study, the contrast maps revealed a lot of variability across individuals. Some participants showed even the exact opposite patterns of activation to each other in the way their oscillatory activity reacted to the contrast between the two conditions (see Figures A.1 and A.2 for AT–FA). This highlights the remarkable individual variability in the continuous MEG activation underlying these conditions. It may also be one reason why it has appeared difficult to build group‐level classifiers for the purpose of brain–computer interfaces with this type of data, as we attempted using these same recordings in two previous studies (Zhigalov et al., 2019; Zhu et al., 2023). Similar large individual differences have been witnessed before in the mind‐wandering research (Golchert et al., 2017). Our results demonstrate the strength of using contrasts among conditions, rather than treating each condition separately, for achieving behaviorally (and individually) meaningful information from ongoing oscillatory activity. From the three different condition pairs, we found significant associations to behavioral trait variables only in the AT–FA contrast. In the AT condition, higher anxiety scores (BAI) and lower behavioral inhibition scores (BIS) correspond to higher suppression (higher alpha) in the temporal areas and lower suppression (lower alpha) in the precuneus, when compared to the FA condition. Thus, the lack of difference in the precuneus on average when comparing FA to AT might be partly due to differences between individuals (and the behavioral traits) that make the difference average out. The opposite direction of associations with BAI and BIS appears first as somewhat contradictory, as both of these trait questionnaires have been linked with the level of anxiety. However, our results, which were confirmed also by separately correlating the brain measures with each trait measure, give indication that these two questionnaires actually tap on independent characteristics in human experience, at least from the perspective of their cortical correlates. In our data, BIS and BAI were only weakly correlated with a Spearman correlation coefficient .38. Similar results have been reported in an earlier study, where behavioral inhibition and anxious arousal had a correlation coefficient .26 (Campbell‐Sills et al., 2004). Thus, these questionnaires seem indeed to measure different aspects of behavior, and therefore, it is plausible, and highly relevant, to consider brain activation that is simultaneously correlated to one and anticorrelated to the other.

Our study was exploratory in nature, leaving us with an open field of potential outcomes. Given the engagement of both trait anxiety and the AT condition, we could assume, for the purpose of simplification, that the FA serves as a stable baseline. Consequently, the contemplation of anxiety‐inducing scenarios brings forth a correlation between high temporal and low parietal midline activation and high trait anxiety (BAI) and low behavioral inhibition (BIS). As in Figure 4, we could further simplify this interpretation and consider trait variables quasi‐independently. Thus, high trait anxiety correlates with increased temporal activation and reduced parietal midline activation. If we interpret the parietal midline regions as components of the DMN, our results suggest that individuals prone to anxiety show less suppression (lower alpha) in the DMN while contemplating negative imagery. Given the DMN's negative correlation with external attention and positive correlation with remembering, future envisioning, and social inference (Buckner & DiNicola, 2019), this could indicate a more immersive internal experience for anxiety‐prone individuals. However, the role of alpha in non‐sensory areas lacks consensus, rendering this interpretation somewhat speculative (Braboszcz & Delorme, 2011). A similar argument applies to temporoparietal lateral areas. If these areas are related to the control network (FPN), it would suggest less activation (higher alpha) and thus “less control” during the contemplation of anxious imagery for participants with higher trait anxiety.

An intriguing aspect in the results was that the above brain areas tended to react in the opposite way for a person with a higher behavioral inhibition score. Although it is known that behavioral inhibition is associated with an increased risk of developing anxiety disorders (Svihra & Katzman, 2004), it is also argued that high behavioral inhibition can reduce a person's level of fear or negative affect by facilitating the disengagement of attention from negative thoughts (White et al., 2011). Here, by “disengagement,” we refer to a decreased level of attention or immersion in a particular stimulus or task. It is thus possible that the observed pattern of activation reflects a scenario where a high trait of behavioral inhibition is “protecting” the participant from deeply engaging in a task of placing oneself or someone close in a negative situation. Thus, if a person with a tendency to be anxious can on average have a more immersive experience with the negative imagery task, a person with a tendency of inhibition can on average have a more disengaged experience. This reasoning, building on our neuroimaging data, would therefore indicate that a person with a high tendency of anxiety and a low tendency of behavioral inhibition is the most susceptible to a very immersive experience. Further research, especially in the field of clinical and personality psychology, is needed to test this implication, but it highlights the possibilities of cognitive neuroscience and neuroimaging in evoking novel questions and hypotheses also about individual differences and factors influencing mental health. Considering the loose nature of the association, with the state of mind, behavioral pattern, and brain pattern each serving as moving parts, it is plausible to find other interesting interpretations. Nonetheless, the key takeaway is that it is indeed possible to discover meaningful associations between dispositional traits and neural activation via innovative state manipulations.

### 4.3 Methodological considerations

On the methodological side, the analysis was based on spatial contrast maps, which are band‐ and participant‐specific source space representations of the difference between two conditions. Computing the difference between conditions removes unnecessary variation and noise, as the second condition basically acts as baseline. This opens a window to task‐related activations that would otherwise be hidden in a task‐unrelated activity. We think this is one main reason why we were able to find a connection between traits and states, which has been notably hard in resting state analysis (Dubois et al., 2018). Another key methodological choice was the use of penalized CCA in investigating the associations between the spatial contrast data and the psychological trait data. We altered the standard CCA procedure with two changes: The analysis was made more robust to outliers with rank transformation. To make the analysis more interpretable and less prone to overfitting, we regularized the model with two different penalties: a sparsity imposing L1‐penalty for the trait variables and a L2‐penalty for the contrast variables. Fitting most of the data in a single model makes it possible to find intricate connections in a statistically reliable way. Even if in this study the number of variables in the second set was relatively small, the same methodology can be applied to larger datasets with a lot more variables. To our knowledge, this is the first neuroscientific study utilizing the recent iterative least squares formulation of CCA, which allows adding different convex penalties to the two sets of variables (Mai & Zhang, 2019).

As there exists a body of literature on personality traits studied with EEG (Kuper et al., 2019; Mathersul et al., 2008), we note a few key differences that our study exhibits. First, we employed MEG, which emphasizes different neural sources than EEG and is also considered more spatially accurate as the magnetic field passes through the head without distortion (Singh, 2014). Second, many of the studies use group comparison methods and thus do not explicitly model the individual differences, which is in contrast with the correlation methods used in our study. Third, instead of focusing on asymmetry scores as is often done in earlier studies, we used statistically efficient data‐driven methods to locate the areas of interest, which can also capture asymmetry dynamics if present. Finally, and perhaps most importantly, previous studies are usually based on resting‐state data. In this study, we used data collected with a novel paradigm actively engaging participants in states such as FA or anxiety. The idea of using these more active naturalistic conditions as a possibility to tap into personality traits can have substantial benefits and should be investigated further.

### 4.4 Limitations

There are some limitations in the conducted study. As the analysis was more exploratory than hypothesis‐based, some of the interpretations are naturally speculative. For example, we used the interpretation that increased power of alpha oscillations signifies inhibition in the relevant cortex, even though in the context of internal mentation, power of alpha oscillations is often positively correlated with the intensity of the task (Ceh et al., 2020; Knyazev, 2013). However, it is possible that this positive correlation relates either to an inhibition in a nearby area, or inhibition of an overlapping function in the same area (Klimesch, 2012). In our case, it was not clear beforehand what to expect as regards the alpha power. For example, FA has been associated with both increases and decreases of alpha oscillations in the previous studies (Lomas et al., 2015). Some of the discrepancy could be explained with lower spatial localization of EEG, especially in the older studies, and also with the vague nature of resting state contrast. This study contributes to the existing literature with better localization of MEG and the more well‐defined contrasts yet natural ongoing brain states. It should also be noted that the aperiodic component (1/*f*) of the power spectra was not removed. Some of the results attributed to alpha or beta oscillations could thus also reflect differences in the aperiodic component (Gyurkovics et al., 2022). In our study, we found that the beta oscillations followed the behavior of alpha oscillations quite closely. Yet, in some studies, the association between alpha and beta activations has been found divergent (see, e.g., Groot et al. (2021)). We assume that reason lies in experimental design (note that also we found divergent activations in CCA), but the effect of the aperiodic component should be investigated in a future study.

In this study, we employed three distinct conditions: FA, self‐centered FP, and AT triggered by visual imagery. The latter two were designed to induce a state of deliberate mind wandering, characterized by an “effortful, intentional engagement with unguided thoughts” (Arabacı & Parris, 2018; Golchert et al., 2017), which overlaps with the concept of “focused daydreaming” (Dorsch, 2015), where individuals actively direct their thoughts around a particular theme or subject. Although these induced states might not fully mirror spontaneous, unintentional mind wandering, they offer precise contrasts—neutral and anxious busy mind states—to FA, providing a richer context than traditional resting state data and facilitating a more extensive and nuanced collection of data compared to conventional probe‐based and self‐report methods. However, although the anxious state was specifically constructed as negative, the neutral state was less defined, potentially leading to greater individual variability (Bø et al., 2022). An additional consideration involves the role of “self” in the mind‐wandering states. Participants were expressly directed to envision themselves or a close acquaintance in specific anxiety‐inducing scenarios, whereas in the FP condition, they were simply asked to formulate plans for the future. Consequently, the observed differences between these states may partly reflect variations in self‐related processing, although the FP condition could feasibly involve self‐processing as well. In light of the significant emphasis on mind wandering inherent in our task contrasts, assessing our participants’ innate tendencies for mind wandering could have provided additional insight. Utilizing questionnaires such as the Mind‐Wandering Questionnaire (MWQ) or Daydreaming Frequency Scale (DDFS) could have allowed us to evaluate the potential influence of natural mind‐wandering propensities on the neural correlates, trait characteristics, and self‐reported focus levels during the recordings.

One area of uncertainty arises from our observations related to the bilateral difference in the temporal areas between the AT and the FP conditions. The nature of the images used in the FP condition, while intended to be neutral, might introduce emotional variability among participants based on individual experiences and associations. This complicates our interpretations, particularly when considering the bilateral differences observed. Although we offered interpretations based on known neural connections and functions, these suppositions remain speculative due to the lack of prior literature on the topic.

One important limitation in this methodological approach is that when we contrast these delicate tasks, it is not easy to untangle them without domain information. In the AT–FA contrast, what we interpret as an effect of increase/decrease in mind wandering might as well be an effect of decrease/increase in FA. However, these task contrasts may still give superior information when compared to unconstrained resting state as the contrast, and the difference between the two conditions is informative even without establishing the definite functional role, especially when connected with individual trait characteristics. Another limitation is related to the applicability of the methodological approach. It can only be applied to datasets that contain multiple conditions and is not applicable, for example, if only resting state recordings are available. It is also worth noting that activation patterns shared by both conditions, which are lost in the subtraction, may still be significant determinants of individual variation.

The accuracy of inverse transform depends on the model of the brain, which usually is based on individual MRI images. For this study, however, we did not have MRI images for all participants and, instead, used a default template head model from freesurfer package, to which the digitized head was aligned. Because the real shape of individual heads can vary, the accuracy of the inverse transform is slightly reduced, which must be kept in mind when interpreting the results. Moreover, because of the exploratory approach, we did not correct the *p*‐values for multiple comparisons. This was because of the relatively low sample and the novelties of the methodology we wanted to show. We tried to keep the statistical comparisons to minimum, however, and combined as much as possible in a single model. Both the neuroscientific results and the usability of the methods should be confirmed in future studies.

## 5 CONCLUSIONS

In this study, we aimed to clarify the oscillatory patterns in the brain during naturalistic conditions and their association to behavioral traits. We utilized three different conditions: FA, self‐centered FP, and AT evoked by visual imagery. Our task design provides brain data during extended periods of mind wandering, which is hard with only self‐reports and probes, and well‐defined contrasts for the state of FA, which is usually studied only with respect to unconstrained resting state. We demonstrate oscillatory power differences between the three conditions, with the main result being between the FA and the other (mind‐wandering) conditions. The FA condition, when compared to the other conditions, is likely to show higher alpha and beta power in the temporal areas of the left hemisphere and lower alpha and beta power in the parietal areas of the right hemisphere. We also explored the use of CCA as a way to investigate the link between individual differences in the brain data and the behavioral trait data collected with questionnaires. We demonstrated the ability of the method to extract behaviorally relevant, participant‐specific characteristics of brain activity. In a condition designed to evoke AT relative to FA condition, a pattern of high alpha power in bilateral temporal areas and low alpha power in bilateral precuneus was correlated with BAI scores, representing high tendency to somatic anxiety symptoms, and anticorrelated with BIS scores, mainly representing worry and avoidance of unpleasant situations. Exploration of individual differences through neuroimaging methods has been an increasing trend in neuroimaging studies. Our results provide evidence for the relevance of the ongoing brain activation for behavioral trait characteristics, specifically for the oscillatory dynamics underlying the dimension between focused versus wandering mind. We further demonstrate the utility of a specific methodological approach for approaching individual differences in the context of continuous and naturalistic task settings.

## AUTHOR CONTRIBUTIONS

**Erkka Heinilä**: Data curation; methodology; software; visualization; writing—original draft; writing—review and editing. **Aapo Hyvärinen and Lauri Parkkonen**: Conceptualization; funding acquisition; methodology; supervision; writing—review and editing. **Tiina Parviainen**: Conceptualization; funding acquisition; project administration; supervision; writing—review and editing.

## CONFLICT OF INTEREST STATEMENT

The authors declare that there are no conflicts of interest.

### PEER REVIEW

The peer review history for this article is available at https://publons.com/publon/10.1002/brb3.3428.

## Supporting information

Figure A.1 Individual spatial contrast maps of alpha band in AT–FA condition pair for participants 1–14. Red means that the average power of oscillations during the anxious thoughts condition is higher than the average power of oscillations during the focused attention condition. The blue means the opposite.
Figure A.2 Individual spatial contrast maps of alpha band in AT–FA condition pair for participants 15–28. Red means that the average power of oscillations during the anxious thoughts condition is higher than the average power of oscillations during the focused attention condition. The blue means the opposite.
Figure A.3 Individual spatial contrast maps of alpha band in FP–FA condition pair for participants 1–14. Red means that the average power of oscillations during the future planning condition is higher than the average power of oscillations during the focused attention condition. The blue means the opposite.
Figure A.4 Individual spatial contrast maps of alpha band in FP–FA condition pair for participants 15–28. Red means that the average power of oscillations during the future planning condition is higher than the average power of oscillations during the focused attention condition. The blue means the opposite.
Figure A.5 Individual spatial contrast maps of alpha band in AT–FP condition pair for participants 1–14. Red means that the average power of oscillations during the anxious thoughts condition is higher than the average power of oscillations during the future planning condition. The blue means the opposite.
Figure A.6 Individual spatial contrast maps of alpha band in AT–FP condition pair for participants 15–28. Red means that the average power of oscillations during the anxious thoughts condition is higher than the average power of oscillations during the future planning condition. The blue means the opposite.
Figure A.7 The *t*‐values that the permutation cluster tests were based on. The values are computed voxelwise with the participants as samples. The borders of the resulting significant clusters are overlaid on top of the maps.
Figure A.8 Cross validation results for selection of L2‐penalty for AT–FA condition pair. For the selection of the L2‐penalty parameter, we used stochastic cross validation. We had an equidistant grid of 300 sensible parameter values, and for each of them, we randomly split the data into 2 14‐participant samples for 2000 times. In each case, we used the first sample (the training set) to fit the CCA model, and then using the canonical weights of the first canonical correlation, computed correlation in the other sample (the test set). In the end, the correlation coefficients over the 2000 splits were averaged. This procedure results in a score (the average correlation in the test set) for each L2‐penalty. The best performing penalty (shown in red) was 1.475.
Figure A.9 Means and deviations of participants’ answers to how they *felt* in the previous two miniblocks (of the same condition) organized by the session and the condition.
Figure A.10 Means and deviations of participants’ answers to how focused they were in the previous two miniblocks (of the same condition) organized by the session and the condition.
Figure A.11 Scree plot for the explained variance of the principal components in the alpha‐only CCA analysis of the AT–FA contrast and the trait data. The number of components was not selected using the plot, but the plot shows that most of the variation is captured in the four principal components, and the additional variance gained by including more components is low.
Figure A.12 To investigate the potential influence of meditation experience (in years) on the results, we conducted a supplementary analysis adopting an alpha‐only CCA for the AT–FA condition. In this approach, meditation experience was used as a predictor to estimate its influence on each variable separately (four each for brain and behavior). Subsequently, the penalized CCA analysis was performed using the residuals instead of the original variables, effectively controlling for the potential influence of meditation experience. The observed similarity between these supplementary results and the main findings suggests that meditation experience did not significantly confound the outcomes. We acknowledge that this regression approach, while reasonable, might introduce certain biases and, moving forward, advocate for further research to explore more explicit and robust methodologies to incorporate covariates like meditation experience, potentially paving the way for more nuanced analyses in future studies.

## Data Availability

Data will be made available upon request, taking into account the current legislation and ethics regulations in Finland. In this case, a formal data sharing agreement will be needed, and data has to be fully anonymized before sharing.
