# Supplementary material for: Penalized canonical correlation analysis reveals a relationship between temperament traits and brain oscillations during mind wandering
Source: Brain Behav. 2024 Feb 15;14(2):e3428. doi: 10.1002/brb3.3428 (PMC10869894; doi:10.1002/brb3.3428)
Supplement: Supplementary file 1 — Figure A.1 Individual spatial contrast maps of alpha band in AT–FA condition pair for participants 1–14. Red means that the average power of oscillations during the anxious thoughts condition is higher than the average power of oscillations during the focused attention condition. The blue means the opposite. Figure A.2 Individual spatial contrast maps of alpha band in AT–FA condition pair for participants 15–28. Red means that the average power of oscillations during the anxious thoughts condition is higher than the average power of oscillations during the focused attention condition. The blue means the opposite. Figure A.3 Individual spatial contrast maps of alpha band in FP–FA condition pair for participants 1–14. Red means that the average power of oscillations during the future planning condition is higher than the average power of oscillations during the focused attention condition. The blue means the opposite. Figure A.4 Individual spatial contrast maps of alpha band in FP–FA condition pair for participants 15–28. Red means that the average power of oscillations during the future planning condition is higher than the average power of oscillations during the focused attention condition. The blue means the opposite. Figure A.5 Individual spatial contrast maps of alpha band in AT–FP condition pair for participants 1–14. Red means that the average power of oscillations during the anxious thoughts condition is higher than the average power of oscillations during the future planning condition. The blue means the opposite. Figure A.6 Individual spatial contrast maps of alpha band in AT–FP condition pair for participants 15–28. Red means that the average power of oscillations during the anxious thoughts condition is higher than the average power of oscillations during the future planning condition. The blue means the opposite. Figure A.7 The t‐values that the permutation cluster tests were based on. The values are computed voxelwise with the participants as samples. T [file BRB3-14-e3428-s001.docx]

## Appendix. Supplementary figures.


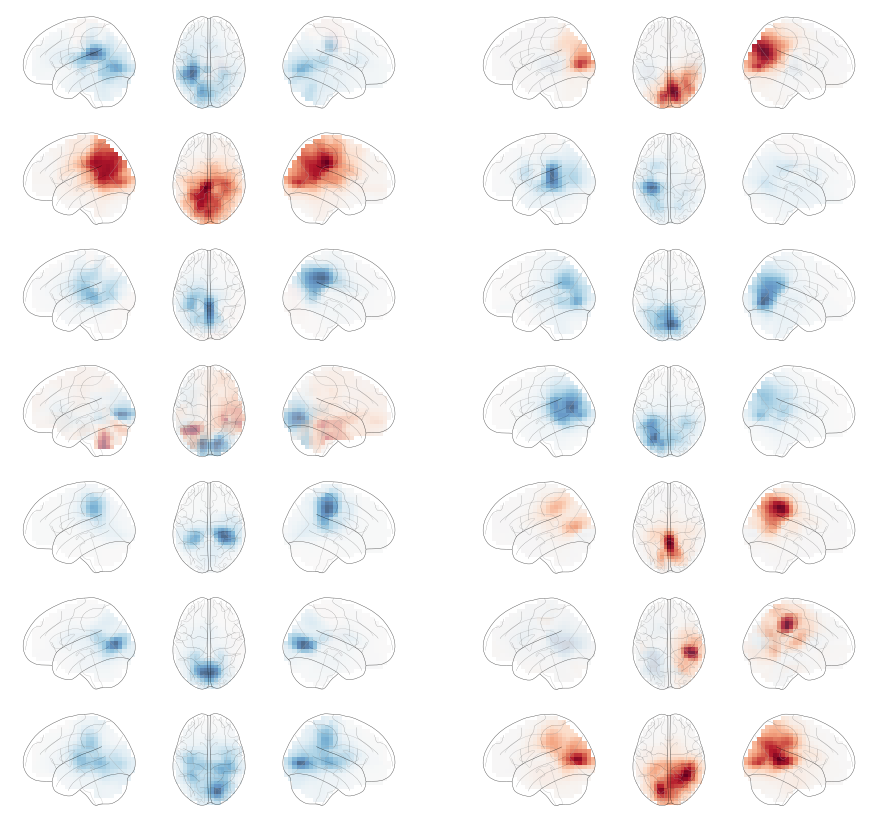


**Fig A.1. Individual spatial contrast maps of alpha band in AT–FA condition pair for participants 1-14. The red color means that the average power of oscillations during the anxious thoughts condition is higher than the average power of oscillations during the focused attention condition. The blue means the opposite.**

**
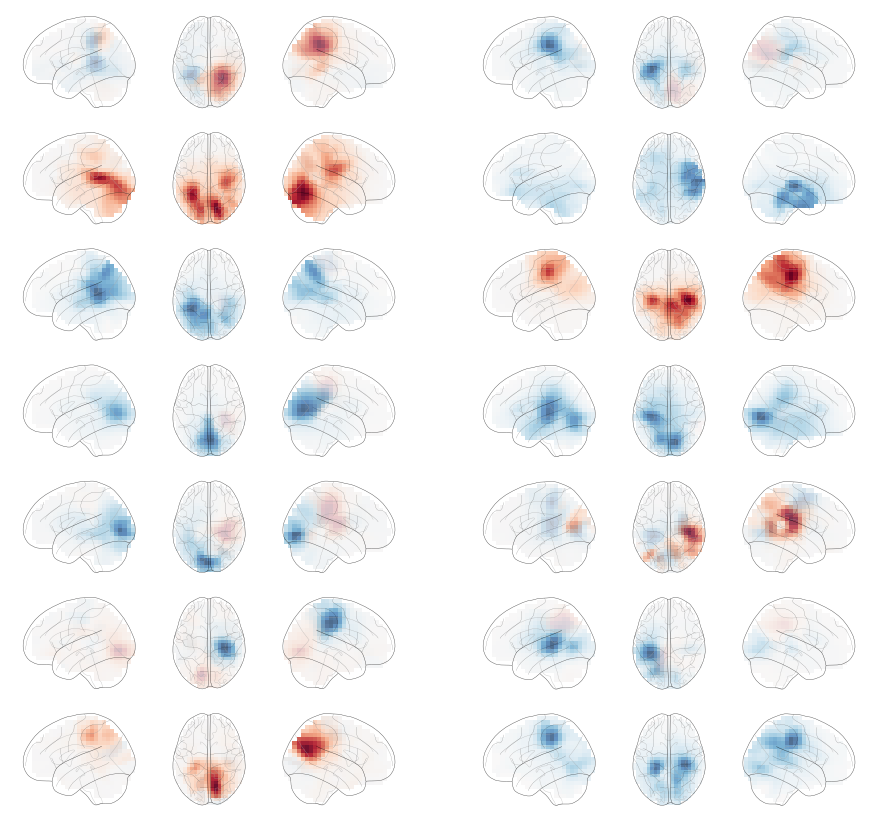
**

**Fig A.2. Individual spatial contrast maps of alpha band in AT–FA condition pair for participants 15-28. The red color means that the average power of oscillations during the anxious thoughts condition is higher than the average power of oscillations during the focused attention condition. The blue means the opposite.**

**
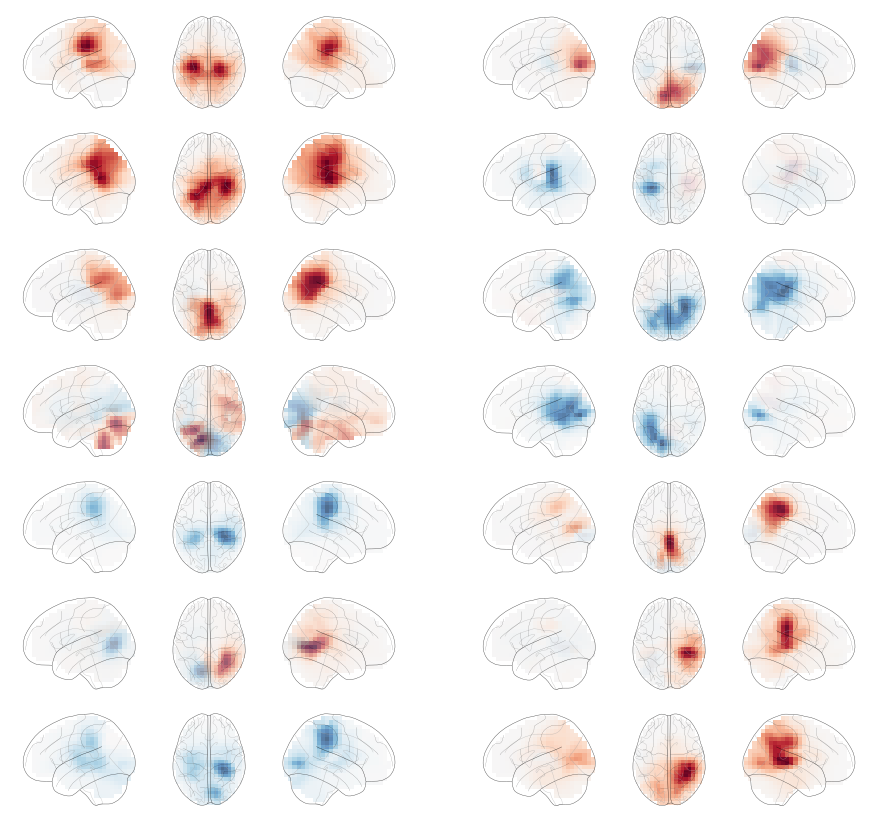
**

**Fig A.3. Individual spatial contrast maps of alpha band in FP–FA condition pair for participants 1-14. The red color means that the average power of oscillations during the future planning condition is higher than the average power of oscillations during the focused attention condition. The blue means the opposite.**

**
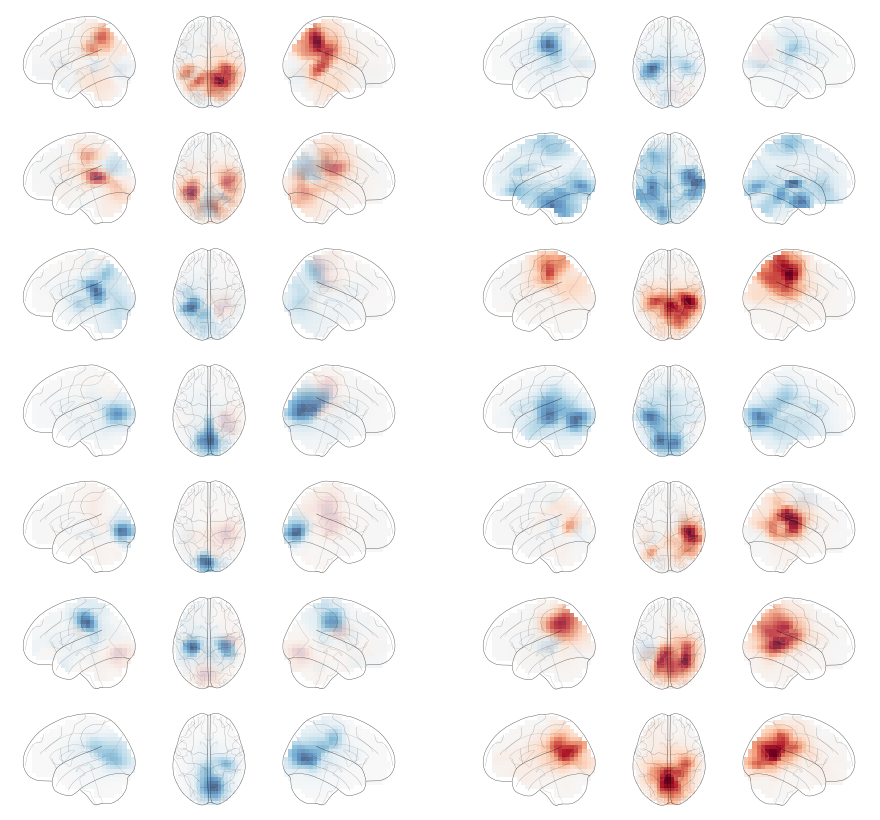
**

**Fig A.4. Individual spatial contrast maps of alpha band in FP–FA condition pair for participants 15-28. The red color means that the average power of oscillations during the future planning condition is higher than the average power of oscillations during the focused attention condition. The blue means the opposite.**

**
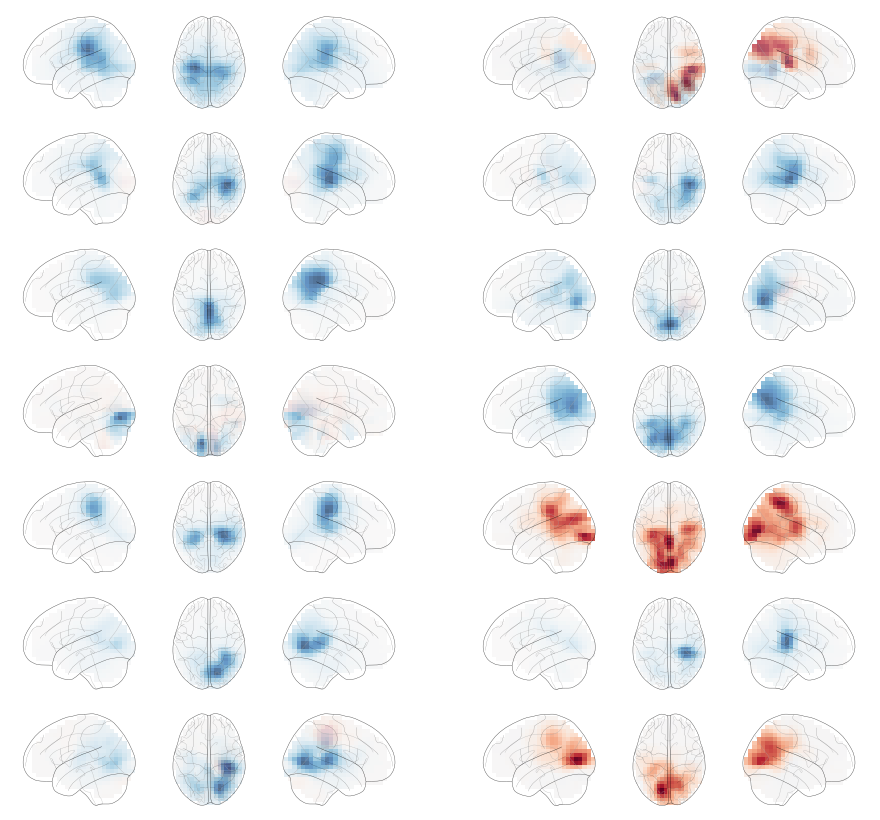
**

**Fig A.5. Individual spatial contrast maps of alpha band in AT–FP condition pair for participants 1-14. The red color means that the average power of oscillations during the anxious thoughts condition is higher than the average power of oscillations during the future planning condition. The blue means the opposite.**

**
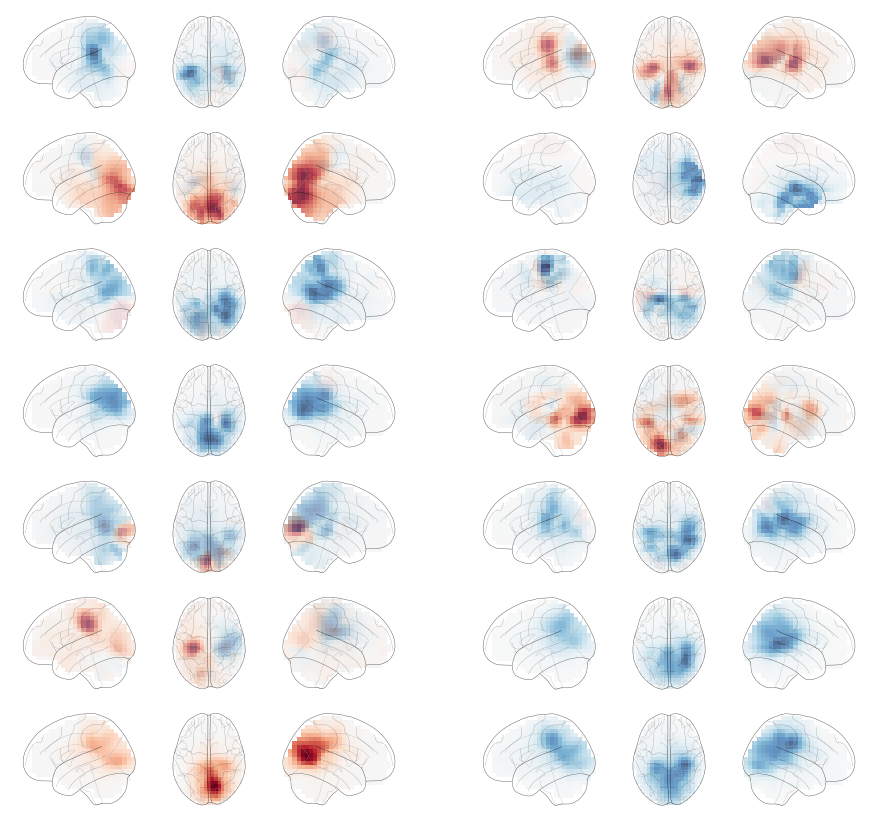
**

**Fig A.6. Individual spatial contrast maps of alpha band in AT–FP condition pair for participants 15-28. The red color means that the average power of oscillations during the anxious thoughts condition is higher than the average power of oscillations during the future planning condition. The blue means the opposite.**


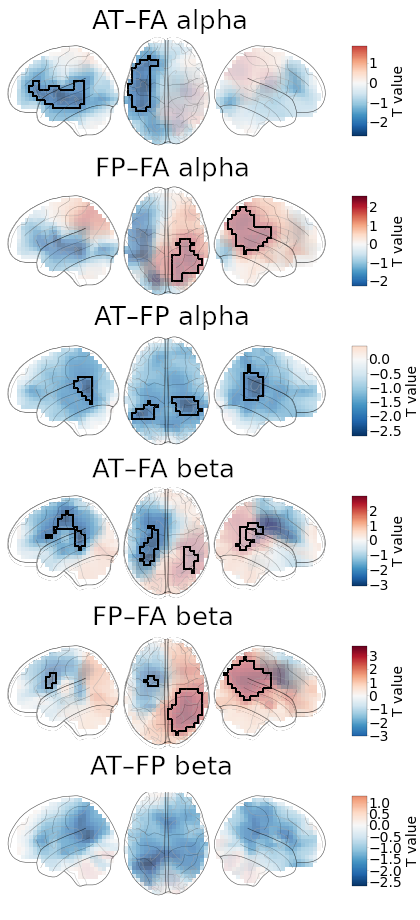


**Fig A.7. The t-values that the permutation cluster tests were based on. The values are computed voxelwise with the participants as samples. The borders of the resulting significant clusters are overlaid on top of the maps.**

**
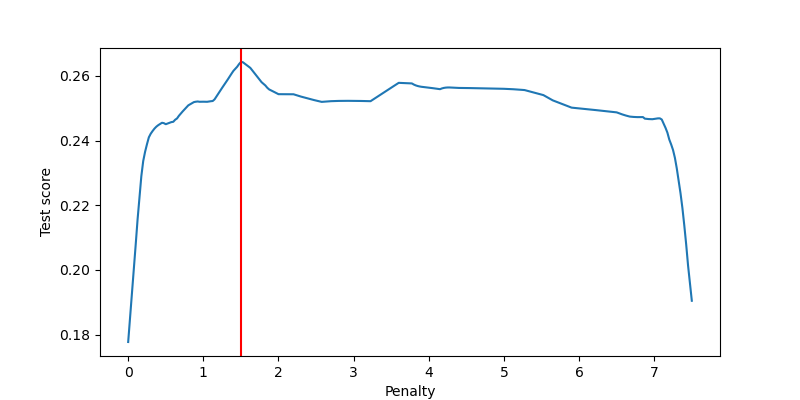
**

**Fig A.8. Cross validation results for selection of L2-penalty for AT–FA condition pair. For the selection of the L2-penalty parameter, we used stochastic cross validation. We had an equidistant grid of 300 sensible parameter values, and for each of them we randomly split the data into two 14-participant samples for 2000 times. In each case, we used the first sample (the training set) to fit the CCA model, and then using the canonical weights of the first canonical correlation, computed correlation in the other sample (the test set). In the end, the correlation coefficients over the 2000 splits were averaged. This procedure results in a score (the average correlation in the test set) for each L2-penalty. The best-performing penalty (shown in red) was 1.475.**

**
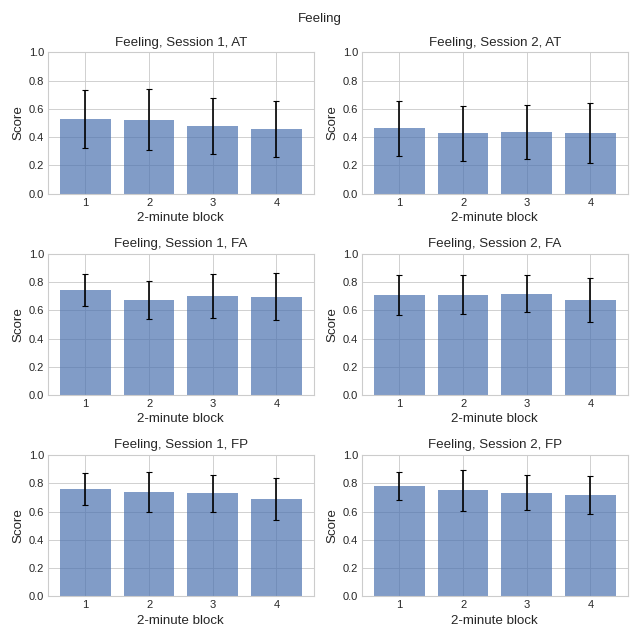
**

**Fig A.9. Means and deviations of participants’ answers to how they *felt* in the previous two miniblocks (of the same condition) organized by the session and the condition.**

**
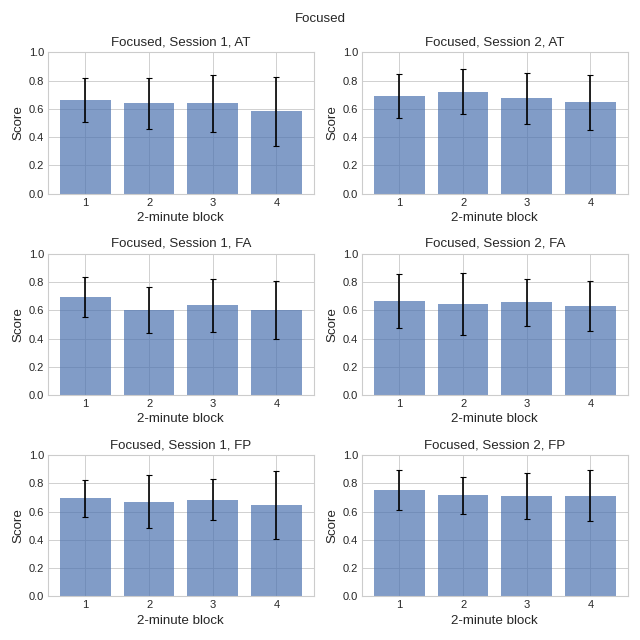
**

**Fig A.10. Means and deviations of participants’ answers to how focused they were in the previous two miniblocks (of the same condition) organized by the session and the condition.**

**
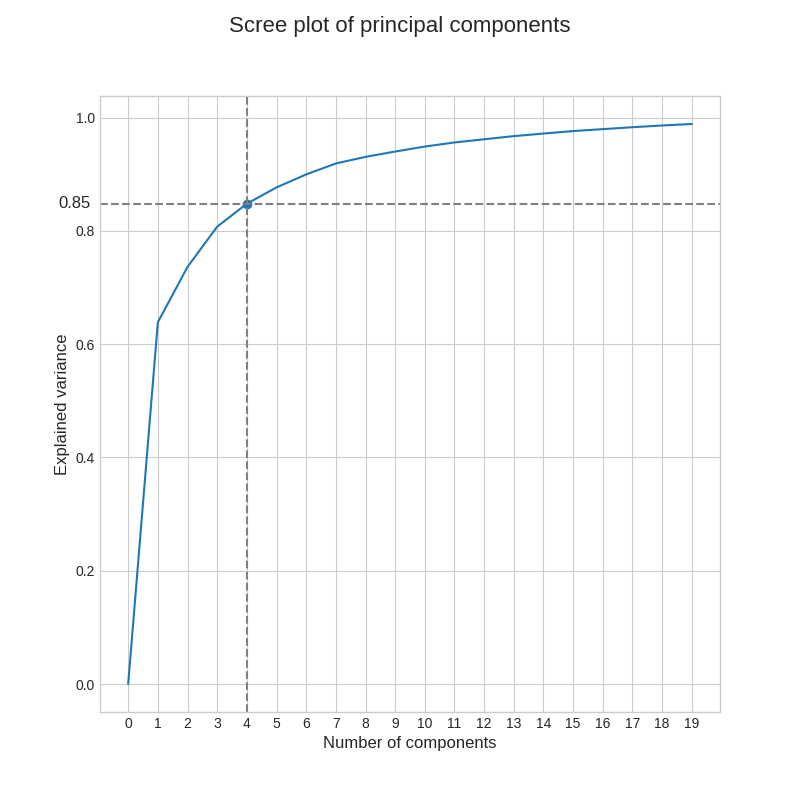
**

**Fig A.11. Scree plot for the explained variance of the principal components in the alpha-only CCA analysis of the AT-FA contrast data and the trait data. The number of components was not selected using the plot, but the plot shows that most of the variation is captured in the four principal components and the additional variance gained by including more components is low.**

**
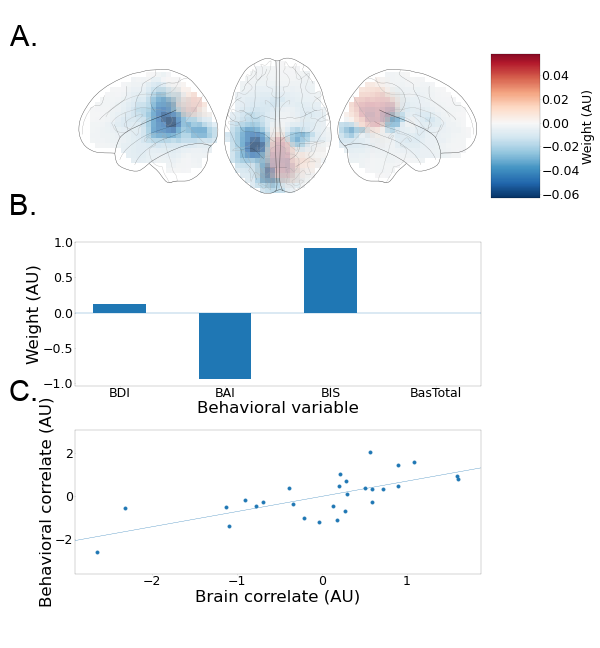
**

**Fig A.12. To investigate the potential influence of meditation experience (in years) on the results, we conducted a supplementary analysis adopting an alpha-only CCA for the AT-FA condition. In this approach, meditation experience was used as a predictor to estimate its influence on each variable separately (four each for brain and behavior). Subsequently, the penalized CCA analysis was performed using the residuals instead of the original variables, effectively controlling for the potential influence of meditation experience. The observed similarity between these supplementary results and the main findings suggests that meditation experience did not significantly confound the outcomes. We acknowledge that this regression approach, while reasonable, might introduce certain biases and, moving forward, advocate for further research to explore more explicit and robust methodologies to incorporate covariates like meditation experience, potentially paving the way for more nuanced analyses in future studies.**
